# Arctic Health in Russia

**DOI:** 10.3402/ijch.v72i0.20724

**Published:** 2013-03-19

**Authors:** Kue Young

It is often pointed out that the Russian Federation accounts for almost half of the circumpolar world. Yet information on health issues in Arctic Russia is not widely available in the English language. The *International Journal of Circumpolar Health* considers its special responsibility to publish articles about Russia and written by our colleagues in Russia. In 2012, of 83 published articles (excluding editorials and letters), only 6 were from Russia. A few years ago, we also published a supplement entitled *Indigenous Peoples of Northern Russia: Anthropology and Health* by Kozlov, Vershubsky and Kozlova (www.circumpolarhealthjournal.net/public/journals/32/chs/CHS_2007_1.pdf).

It is timely that we are now publishing the series of 6 articles by Alexey A. Dudarev and his colleagues from the Northwest Public Health Research Centre in St. Petersburg, in collaboration with Jon Øyvind Odland of the University of Tromsø, Norway. They cover two topics: occupational health and cancer. These articles are based on literature reviews, personal observations and analyses of official statistics.

In the 3 articles on occupational health, the authors paint a grim picture of widespread disregard of health and safety regulations, poor enforcement, and gross under-reporting of occupational diseases and injuries. It is a national problem, one which appears to be aggravated in some Arctic regions, where extensive natural resource development projects are underway.

The remaining three articles focus on the Chukotka Autonomous Okrug, located in the northeastern-most part of the country. The first article provides an interesting overview of the history of the region, especially its economic development, and its impact on the health of the people. This is followed by an article on the overall epidemiology of cancer in the region, and an article on cancer mortality among the indigenous population of a coastal district within the region. Since information on the pattern of cancer in Russia is very limited – the St. Petersburg registry is the only Russian registry included in the World Health Organization's *Cancer Incidence in Five Continents* – these articles on cancer in Chukotka fill an important gap of our knowledge.

Among the circumpolar countries, Norway has played a very important role in providing technical assistance to post-Soviet Russia and promoting collaborative projects in research and education. The University of Tromsø in particular has taken a leadership role. This Special Issue would not have been published without the financial assistance provided by our colleagues in Tromsø, to whom we extend our deepest appreciation.

*Kue Young* Editor-in-Chief

